# *Primula exserta* (Primulaceae), a new species in *Primula* sect. *Aleuritia* from Yunnan, China

**DOI:** 10.3897/phytokeys.278.202553

**Published:** 2026-08-14

**Authors:** Li Yang, Gui-Liang Zhang, Yang-Yang Zheng, Qing-Qing Ye, Xin Tan, Lei Cai, Zhi-Kun Wu

**Affiliations:** 1 Department of Pharmacy, Guizhou University of Traditional Chinese Medicine, Guiyang, 550025, Guizhou, China Yunnan Key Laboratory for Integrative Conservation of Plant Species with Extremely Small Populations/ Key Laboratory for Plant Diversity and Biogeography of East Asia, Kunming Institute of Botany, Chinese Academy of Sciences Kunming China; 2 Hekou Branch of Managent and Protection Bureau of Daweishan National Nature Reserve, Hekou, 661399, Yunnan, China Department of Pharmacy, Guizhou University of Traditional Chinese Medicine Guiyang China; 3 Yunnan Key Laboratory for Integrative Conservation of Plant Species with Extremely Small Populations/ Key Laboratory for Plant Diversity and Biogeography of East Asia, Kunming Institute of Botany, Chinese Academy of Sciences, Kunming, 650201, Yunnan, China Hekou Branch of Managent and Protection Bureau of Daweishan National Nature Reserve Hekou China

**Keywords:** Conservation status, diversity, nomenclature, *
Primula
*, taxonomy, Yunnan

## Abstract

*Primula exserta* Lei Cai, Gui L. Zhang & Z. K. Wu, a new species of Primulaceae from Yunnan, China, is described and illustrated. Morphological evidence supports its placement within *Primula* sect. *Aleuritia*, a section characterized by entirely glabrous plants that lack scales at the base of the leaf rosette, has a moderate to small habit and typically bears farina on the foliage and scape. The new species differs from other members of this section by its petiole with broad wings, 7–8-lobed corolla and calyx, corresponding 7–8 stamens, and elongated filaments and a style exserted far beyond the mouth of the corolla tube. Information on its distribution, a morphological comparison with related species, and a preliminary assessment of its conservation status are also provided.

## Introduction

The genus *Primula* L. represents the largest genus within the family Primulaceae, comprising approximately 556 species worldwide (POWO 2026). It is considered a typical example of an alpine plant genus and an excellent ornamental group and is also recognized as one of the representative taxa of young, rapidly radiating lineages among angiosperms. Species of the genus are mainly distributed in temperate and alpine regions of the Northern Hemisphere, with only a few species occurring in the mountains of Africa (Ethiopia), tropical Asia (Java and Sumatra), and South America (Hu 1990, 1994; Hu and Kelso 1996). China harbors 358 species of *Primula* (Liu 2026), which are classified into 24 sections. Southwest China, particularly the Eastern Himalayas and the Hengduan Mountains region, serves as the modern center of distribution and diversity for this genus, with the majority of species concentrated in western Sichuan, southeastern Xizang, and northwestern Yunnan.

*Primula* sect. *Aleuritia* represents a substantial group within the genus *Primula*. Across its broad distribution range, species of this section occur throughout nearly the entire range of the genus, extending from the circumpolar region to the major mountain systems of Europe, North America, and Asia (Hu 1990, 1994; Hu and Kelso 1996). Notably, this is the only section within *Primula* that includes representative species in South America (Hu 1994; Basak et al. 2014). According to the Flora Republicae Popularis Sinicae, 46 species of sect. *Aleuritia* are distributed in China (Hu 1990). Since the publication of the Flora Republicae Popularis Sinicae, extensive field investigations conducted over the past three decades have led to the description of eight additional species (Basak and Maiti 2001; Wu et al. 2013; Xu et al. 2015, 2017, 2019; Ju et al. 2023; Wu et al. 2023a; Yang et al. 2024). To date, the number of species of sect. *Aleuritia* recorded in China has reached 55.

Yunnan is one of the biodiversity hotspots in China, characterized by diverse habitats ranging from tropical to alpine tundra zones. It harbors 176 species of the genus *Primula* (Yang et al. 2023a). With intensified field surveys in recent years, six new species have been discovered (Wu et al. 2023a, 2023b; Yang et al. 2023b, 2023c; Ru et al. 2026). Currently, Yunnan has a total of 182 species of *Primula*.

In April 2021, during a botanical survey of the Dawei Mountains in Yunnan Province, China, a morphologically distinctive population of *Primula* was encountered. The plants possess obovate to spatulate leaves covered with yellow farina and a small habit and lack scales at the base of the leaf rosette; the entire plant is glabrous. These characteristics indicate its placement within *P.* sect. *Aleuritia*. Nevertheless, several features distinguish it from all known species of the section, including a cup-shaped calyx with 7–8 lobes divided almost to the base, a campanulate corolla with 7–8 lobes, uniform floral morphology, and stamens and a style distinctly exserted far beyond the mouth of the corolla tube. In subsequent years (2022–2026), this area was continuously surveyed during the flowering period, and only one additional population was located. The floral traits of these primroses proved to be stable. Following a thorough examination of the relevant literature and herbarium specimens, this plant was concluded to represent a species new to science within *P.* sect. *Aleuritia*. Accordingly, it is described and illustrated here, and an assessment of its conservation status is provided.

## Materials and methods

Based on specimens and living plants collected from Hekou and Pingbian in Yunnan Province, morphological observations, measurements, and descriptions were conducted on 15 randomly selected living individuals per population. Traits assessed included growth habit, rhizome morphology, the presence of farina, leaf characteristics, calyx and corolla structure, and flower color. Comparative morphological analyses of allied species were conducted by examining living plants from their type localities and herbarium specimens from the online herbarium of the Royal Botanic Garden Edinburgh (E) and the online database of the Plant Science Data Centre (https://www.plantplus.cn/cn), as well as by reviewing the relevant literature (Hu 1990, 1994; Hu and Kelso 1996; Wu et al. 2013; Xu et al. 2017). Key morphological characters of living plants from the type locality of *P. exserta* were measured using digital calipers. The morphological characters of *Primula membranifolia* Franch., *Primula mianyangensis* G. Hao & C.M. Hu, and *Primula pengzhouensis* C.M. Hu, G. Hao & Y. Xu were obtained from living plants collected from their respective type localities and from their original descriptions (Hu 1990; Wu et al. 2013; Xu et al. 2017). The conservation status of the new species was assessed following the guidelines of the IUCN Red List Categories and Criteria (IUCN Standards & Petitions Committee 2024).

## Taxonomic treatment

### Primula exserta

Taxon classificationPlantaeEricalesPrimulaceae

Lei Cai, Gui L.Zhang & Z.K.Wu sp. nov.

3E748DE0-E16E-565F-8A42-F94B8C8F5D26

urn:lsid:ipni.org:names:77394089-1

**Figure 1.**
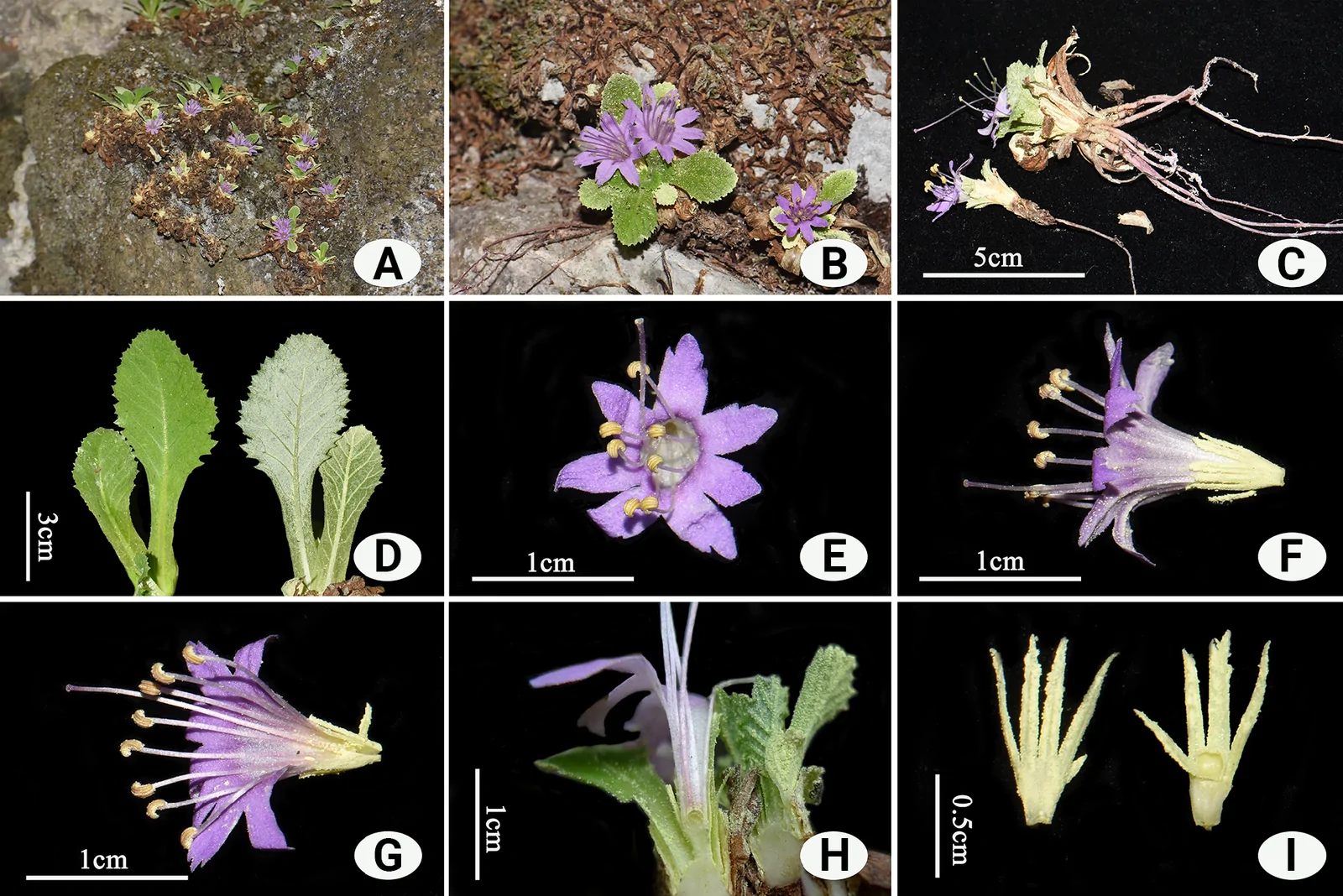
*Primula exserta* sp. nov. **A**. Habitat; **B**. Habit during flowering; **C**. Lateral view of the plant; **D**. Leaves, left: upper surface, right: lower surface; **E**. Flower, frontal view; **F**. Flower, lateral view; **G**. Dissected corolla showing the anthers and stigmas; **H**. Dissected corolla with the ovary; **I**. Dissected calyx.

**Figure 2.**
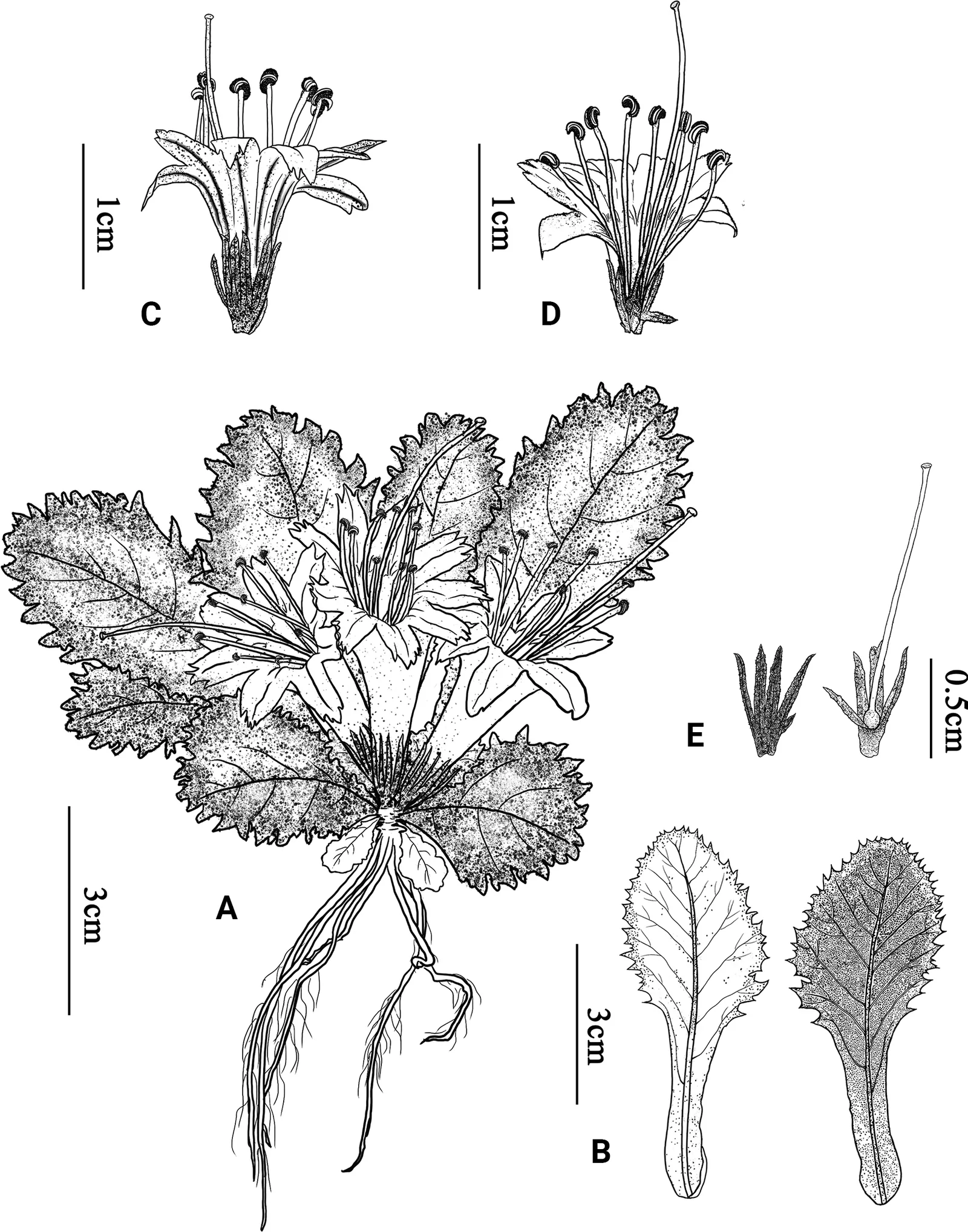
*Primula exserta* sp. nov. **A**. Flowering habit; **B**. Leaves, left: upper surface, right: lower surface; **C**. Flower, lateral view; **D**. Dissected corolla showing the anthers and stigmas; **E**. Dissected calyx. Drawn by Ms. Yang-yang Zheng.

**Figure 3.**
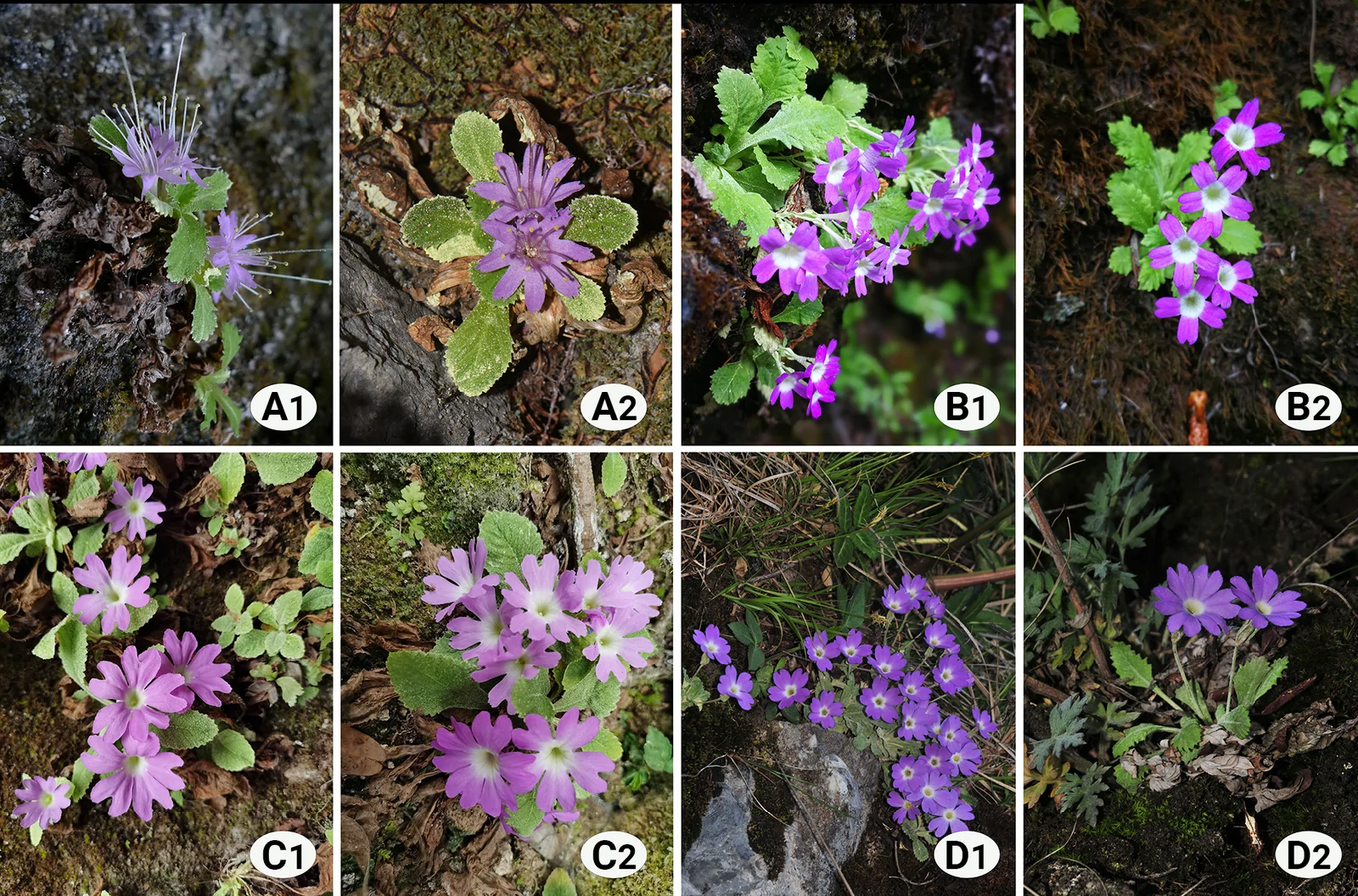
*Primula exserta* sp. nov. and three morphologically similar taxa in *Primula* sect. *Aleuritia*. **A1, A2**. *P. exserta*; **B1, B2**. *P. membranifolia* (Dali, Yunnan, type locality); **C1, C2**. *P. pengzhouensis* (Pengzhou, Sichuan, type locality); **D1, D2**. *P. mianyangensis* (Mianyang, Sichuan, type locality).

Figs 1, 2, Fig. 3: A1, A2

#### Diagnosis.

This new species is most similar to *P. membranifolia*, *P. pengzhouensis*, and *P. mianyangensis* within the same section, all of which share the following characteristics: aboveground parts farinose, calyx campanulate or narrowly campanulate, and leaves obovate or sub-spatulate. However, it is distinguished from the three species mentioned above by the following morphological features: petiole with broad wings, 7–8-lobed corolla and calyx, corresponding 7–8 stamens, floral type uniform: stamens of all flowers inserted at the middle of the corolla tube, free from the tube at the mouth, extending ca. 5 mm beyond the tube, style 2.5–3 cm long, longer than the stamens (Figs 1, 2). A comparison of plant photographs of *P. exserta* with *P. membranifolia*, *P. pengzhouensis*, and *P. mianyangensis* is provided in Fig. 3, and the main morphological differences are summarized in Table 1.

**Table 1.** Morphological comparison of *P. exserta*, *P. membranifolia*, *P. pengzhouensis*, and *P. mianyangensis*.

**Characters**	***P. exserta***	***P. membranifolia***	***P. pengzhouensis***	***P. mianyangensis***
**Leaves blade**	Obovate; apex subrounded; margin densely serrate, occasionally doubly serrate	Elliptic; apex rounded; margin serrate, with obtuse or slightly acute teeth	Elliptic; apex broadly obtuse to subrounded; margin serrate-dentate	Elliptic; apex obtuse to more or less acute; margin dentate-serrate from the lower 1/3
**Petiole**	Broadly winged, nearly as long as the leaf blade	Narrowly winged, 1/2 as long as the leaf blade, rarely nearly as long as the leaf blade	Narrowly winged; inner leaves: petiole 1/3–1/2 as long as the leaf blade; outer leaves: petiole usually as long as the leaf blade	Unwinged; inner leaves: petiole almost as long as the leaf blade; outer leaves: petiole approximately twice as long as the leaf blade
**Scapes**	Almost invisible	(1–)2–5 cm tall	At most 2 mm tall	Almost invisible
**Flower**	Monomorphic; filaments and style conspicuously exserted from the corolla tube and longer than the corolla lobes	Distyly, anthers and filaments all included within the corolla tube	Distyly, anthers and filaments all included within the corolla tube	Distyly, anthers and filaments all included within the corolla tube
**Calyx**	Narrowly campanulate, 3–5 mm long, 7–8-lobed, divided nearly to base	Campanulate, 3–4 mm long, 5-lobed, divided to or slightly beyond the middle	Narrowly campanulate, 5–7 mm long, 5-lobed, parted slightly beyond middle	Narrowly campanulate, 8–9 mm long, 5-lobed, split to 2/3 of its length
**Corolla**	Pale purple or pink; 7–8-lobed, oblong, apex entire or irregularly divided	Pale purplish red; 5-lobed, obovate, apex deeply emarginate	Rose or pale purple, exannulate; 5-lobed, obovate, deeply emarginate	Pale rose-purple; 5-lobed, obovate, bilobed, lobules entire

#### Type.

China • Yunnan: Hekou County, Lianhuatan Township, Zhonglinggang Village, Wanzhangya; 22°55'00"N, 103°39'57"E, 1700 m alt.; 22 May 2026 (fl.); *Lei Cai and Guiliang Zhang CL26052201* (holotype: KUN!; isotypes: KUN!).

#### Description.

Perennial herb. Aboveground parts, except petals farinose; entire plant glabrous. Rhizome short and thick, bearing numerous fleshy roots and fibrous rootlets. ***Leaves*** rosette somewhat compact, arising from the apex of a greatly shortened rhizome, with withered leaves from the previous year remaining at the base; leaf blade obovate, oblanceolate, or sub-spatulate, 1–3 cm long, 0.5–1.5 cm wide, apex subrounded, base gradually attenuate into a wide winged petiole, margin densely serrate, occasionally doubly serrate, membranous when dry, upper leaf surface slightly farinose, lower leaf surface covered with easily deciduous yellow farina; midvein slightly broad, lateral veins slender and prominent on the lower surface; petiole approximately as long as the leaf blade. ***Scapes*** greatly shortened, almost inconspicuous. ***Flowers*** 2–4, borne within the leaf rosette. ***Pedicel*** almost inconspicuous, with a linear bract 2–3 mm long at the base. ***Calyx*** cup-shaped, 3–5 mm long, 7–8-lobed, lobes divided nearly to the base, both inner and outer surfaces heavily covered with yellow farina; lobes narrowly lanceolate, apex acute or acuminate. Flowers monomorphic. ***Corolla*** campanulate, pale purple or pink, throat without an annulus; corolla limb 1.2–1.8 cm in diameter, lobes 7–8, oblong, apex entire or irregularly divided. Corolla tube slender, 1.2–1.5 cm long, externally covered with fine yellow farina; all flowers with 7–8 stamens inserted at the middle of the corolla tube, free from the tube at the mouth, extending ca. 5 mm beyond the tube; style 2.5–3 cm long, longer than the stamens, ovary ovoid, ca. 1 mm long at anthesis. ***Capsule*** unknown.

#### Distribution and habitat.

**Map 1.**
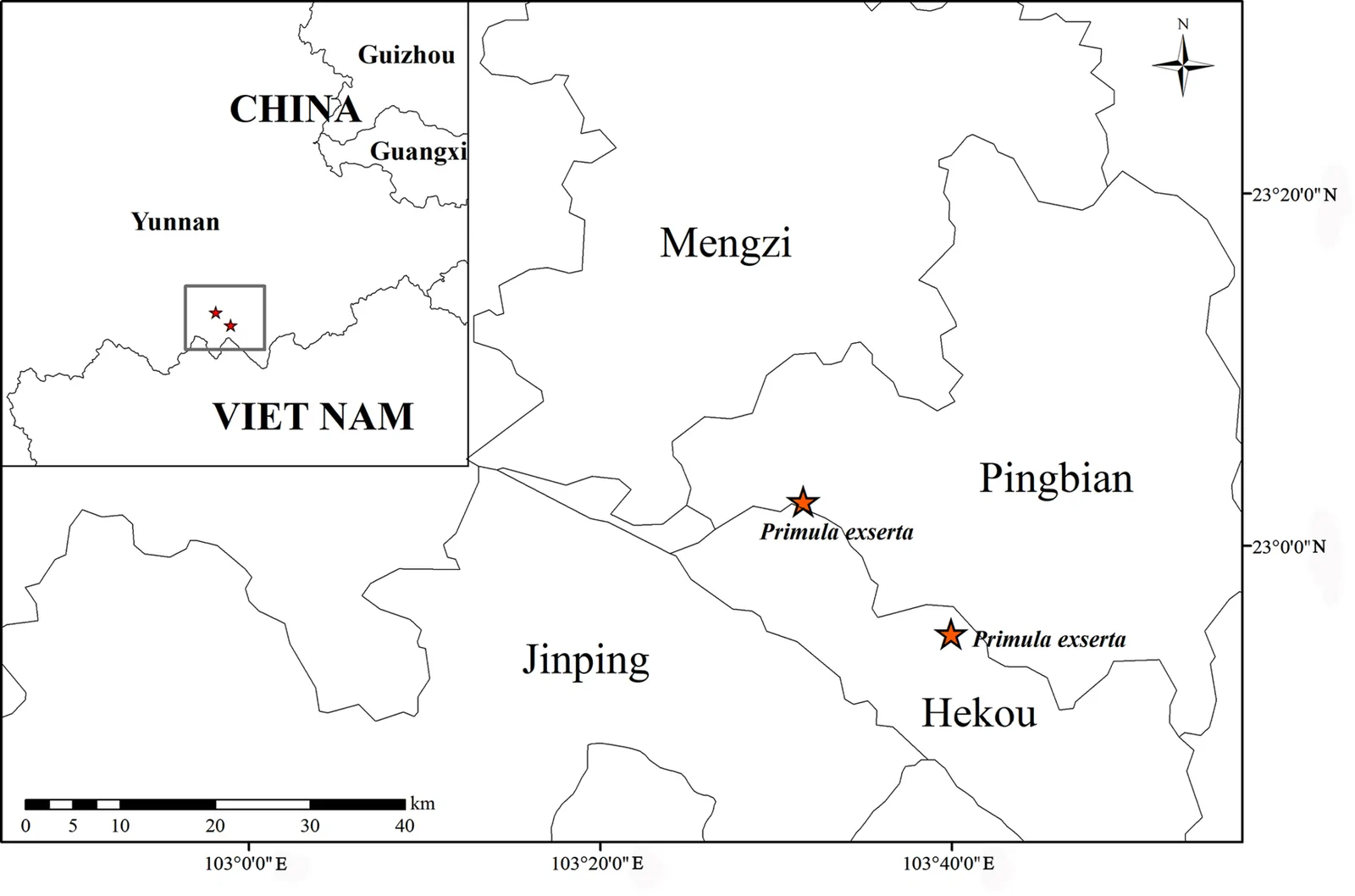
Distribution of *P. exserta* in Hekou and Pingbian, Yunnan.

This new species is currently known from two populations in Zhonglinggang Village, Lianhuatan Township, Hekou County, and Dimi Village, Xinxian Township, Pingbian County, Yunnan Province, China. It is mainly distributed on dry cliffs at elevations between 1700 and 2000 m (Fig. 1; Map 1).

#### Phenology.

The species was observed to flower from April to the end of May.

#### Etymology.

The specific epithet exserta is derived from the Latin *exsertus*, meaning “protruding” or “extended outward,” alluding to the style and stamens that are distinctly exserted beyond the mouth of the corolla tube, a key diagnostic character distinguishing this species from its congeners in *P.* sect. *Aleuritia*.

#### Vernacular name.

Simplified Chinese: 大围山报春; Chinese Mandarin: “dà wéi shān bào chūn.”

#### Additional specimen examine.

China • Yunnan: Pingbian County, Xinxian Township, Dimi Village, Qiche. 23°2'30"N, 103°31'32"E, 1996 m alt.,20 April 2021 (fl.), Lei Cai and Guiliang Zhang *CL21042001* (KUN!).

#### Provisional conservation status.

Since this species was discovered in 2021, only two populations have been found within the Daweishan National Nature Reserve, each consisting of approximately 100 adult individuals. These two localities are in Pingbian County and Hekou County and are separated by a relatively short linear distance. Potential populations may still exist in the surrounding areas. Until further investigations are conducted, the conservation status of this species is assessed as “Data Deficient” (DD) in accordance with the IUCN Red List Criteria.

## Discussion

At first glance, when not in flower, the new species *P. exserta* appears to belong to sect. *Aleuritia* because of its dwarf habit, the presence of farina on the leaves and calyx, and its leaf morphology. However, the distinctive characteristics of its floral morphology—the 7–8-lobed corolla and calyx, corresponding 7–8 stamens, and filaments and style extending beyond the petals—might even lead one to suspect that it represents a new genus. Nevertheless, upon closer examination, it is evident that the filaments are inserted at the middle of the corolla tube, are adnate to the tube, and become free and extend beyond the petals only at the mouth of the tube; the style is also elongated and protrudes well beyond the corolla tube. Based on these floral traits, it is hypothesized that this species may have evolved from a long-styled (pin) flower in which both the filaments and style elongated in response to environmental pressures, thereby facilitating adaptation to a specialized habitat. The precise mechanisms underlying these morphological changes require further confirmation through molecular biological studies. This new species is distributed in southeastern Yunnan Province, a region characterized by a relatively hot climate. In contrast, most species of sect. *Aleuritia* occur in temperate and alpine regions. Among the currently known members of this section, *P. exserta* may represent the southernmost species in the Northern Hemisphere. It is therefore plausible that the specialized floral morphology of this species is a consequence of adaptive evolution following speciation. Instances in which particular floral traits in Primulaceae have deviated from the typical *Primula* morphology and led to recognition at the generic level have occurred previously. For example, the former sect. *Auganthus* has been treated as a distinct genus on three separate occasions, with its distinctive calyx morphology resulting in its recognition under three heterotypic names: *Auganthus* Link, *Oscaria* Lilja, and *Primulidium* Spach (Richards 2002). Furthermore, as noted in previous studies, *Primula luquanensis* Z.K.Wu & Wei Zhou and *Primula nutantiflora* Hemsl., both possessing the fifth type of floral morphology within the genus *Primula*, are currently placed in sect. *Aleuritia* (Wu et al. 2023a). Therefore, based on habit, farinose condition, and leaf morphology, this new species is assigned to sect. *Aleuritia*.

The leaf shape of *P. exserta* resembles that of *P. membranifolia*, but its nearly scapeless habit and distinctive floral structure readily separate it from the latter. In terms of leaf morphology, the absence of a scape, and the presence of farina on both the leaf blades and flowers, *P. exserta* is similar to *P. pengzhouensis* and *P. mianyangensis*. However, it differs from both in having a 7–8-lobed corolla and calyx, corresponding 7–8 stamens, and elongated filaments and a style exserted far beyond the mouth of the corolla tube.

The discovery of this new species enriches the diversity of the genus *Primula* and also provides valuable material for future studies on floral evolution within the genus.

## Supplementary Material

XML Treatment for Primula exserta

## Additional information

### Artificial Intelligence (AI) use

The authors accept full responsibility for the content of the manuscript, including the disclosure of any use of AI.

No AI tools were used in the preparation of this manuscript.

### Author ORCIDs

L. Yang https://orcid.org/0009-0009-2792-6616

G.-L. Zhang https://orcid.org/0000-0003-0501-7533

Y.-Y. Zheng https://orcid.org/0009-0004-0928-5783

Q.-Q. Ye https://orcid.org/0009-0003-7442-4527

X. Tan https://orcid.org/0009-0003-9034-8851

L. Cai https://orcid.org/0000-0002-9251-2745

Z.-K. Wu https://orcid.org/0000-0002-4583-7782

### Data availability

All of the data that support the findings of this study are available in the main text.
